# *In-vitro* Comparison of the Antimicrobial Properties of Glass Ionomer Cements with Zinc Phosphate Cements

**Published:** 2012

**Authors:** Elaheh Vahid Dastjerdie, Mahvash Oskoui, Elham Sayanjali, Fahimeh Sadat Tabatabaei

**Affiliations:** a *Department of Orthodontics, Dental School, Shahid Beheshti University of Medical Sciences, Tehran, Iran.*; b *Department of Microbiology, Pasteur Institute of Iran, Tehran, Iran.*; c *Department of Dental Materials, Dental School, Shahid Beheshti University of Medical Sciences, Tehran, Iran.*

**Keywords:** Antibacterial, Antifungal, Orthodontic bonding, Streptococcus mutans, Candida albicans

## Abstract

White spot lesions are observed in nearly 50% of patients undergoing orthodontic treatment. Long-lasting antibacterial properties of orthodontic cements can reduce this phenomenon. The aim of this research was to compare antimicrobial activity of three commercial glass ionomer cements with three commercial zinc phosphate cements, over time, against *streptococcus mutans* and *candida albicans*. Direct contact test (DCT) was used to evaluate the antibacterial and antifungal activity of products after 48 h and 7 days of incubation. The results demonstrated that all the cements presented antibacterial activity but the antibacterial activity of glass ionomer cements was more than that of zinc phosphate cements. Counts of *C. albicans *after 48 h were lower and statistically different in the GIC group in relation to the control groups. But no differences were observed between GIC and control groups at 7 days. Based on the results of this study, the antimicrobial and mainly antifungal effects of all the cements were so short.

## Introduction

Despite the fact that fixed orthodontic appliances present numerous methods to improve smile esthetics and occlusal relationships, they also pose a challenge to both the patient and the clinician in preserving a healthy dentition. Fixed appliances facilitate the plaque accumulation mainly at the cervical margins of bands and brackets (1). Accordingly, decalcification around the orthodontic brackets and bands, in the form of white spot lesions, is a negative treatment consequence observed in 50% of patients undergoing orthodontic treatment (2-4). *Streptococcus mutans* is one of the bacteria frequently implicated in decalcification of enamel (5-6). In addition, *Candida albicans *may be isolated from the mouth of patients using orthodontic devices (7-10).

Caries prevention in patients using orthodontic appliances is reliant on the control of dental plaque. However, many patients do not take care of their oral hygiene perfectly. Consequently, new research has attempts to develop the dental materials with antibacterial activities one of which are glass ionomer cements that have certain advantages, *e.g.* direct bonding to tooth tissue, thereby avoiding acid etching and cariostatic action due to the fluoride leaching ability (11-12). Orthodontic cements based on glass ionomer have been shown to release fluoride (13). Incorporation of fluorides into the orthodontic cements is based on the conception that fluoride will be released progressively from the set material, so affording continuous long-acting anticariogenic effect (14). It is believed that the fluoride released from the glass ionomer cements contribute the antibacterial activity (15). Different studies have reported the bacteriostatic effect of fluoride ions on oral microorganisms (16).

**Table 1:** Materials evaluated in this study

**Cement type**	**Brand**	**Manufacture**	**Abbreviation**	**Lot no.**
Glass ionomer cement	Resilience	Ortho-Technology, USA	GIC (A)	DP-1/031307
	Band-Tite	American Orthodontics, USA	GIC (B)	070709A
	Ariadent	Apadana Tak, Iran	GIC (C)	GC001
Zinc phosphate cement	Harvard	Harvard Dental GmbH, Germany	ZPC (A)	2112404002
	Hoffman’s	Hoffmann Manufaktur, Germany	ZPC (B)	1404C01
	Ariadent	Apadana Tak, Iran	ZPC (C)	ZF019

Zinc phosphate cement is another material used as an adhesive for orthodontic devices. There is little information available about antibacterial activity of this material. Dahl studied the short acting antibacterial effect of zinc phosphate cement and polycarboxylate and reported that in the agar diffusion test, the zinc phosphate cement exhibited the strongest antibacterial properties (17).

Some methods have been suggested for testing the antimicrobial effect of dental materials. The most frequently employed methods are those based on direct contact test (DCT) (18-19). The direct contact test is a relatively new method that provides the information on the bacterial viability and growth rate and quantitatively measures the effect of direct and close contact between the microorganisms and the tested materials, regardless of the solubility and diffusibility of their components (20).

The growth inhibitory effects of some cements are considered beneficial in preventing bacterial colonization. In addition, the antibacterial activity, during the time, assumes clinical relevance. Based on these outcomes and on the lack of studies on the antibacterial activity of the Iranian orthodontic cements, the aims of this study were as follows:

Comparing the antimicrobial activity of two orthodontic cements against the *streptococcus mutans* and *candida albicans* using DCT, comparing the antimicrobial activity of the cements from different companies, comparing the antibacterial activity during the time.

## Experimental

The orthodontic cements evaluated in this study are shown in Table 1. The antibacterial activity of each material was evaluated against the Streptococcus mutans (ATCC#35668) and Candida albicans (ATCC#10231).

*Preparation of glass ionomer samples*

Six wells (7 mm diameter and 3 mm thickness) were punched in the Muller-Hilton agar plates and filled with 6 cements. A uniform surface was achieved by using a small flat-ended dental instrument, such as a dental spatula. The material was allowed to set in accordance with the manufacturer›s recommendation.

*Antibacterial activity test*

Bacterial strain from stock cultures was cultivated in Brain Heart Infusion broth (Difco, Detroit, USA) at 37^°^C, for 24 h. The top 4 mL of the resulting undisturbed bacterial cultures were transferred to new test tubes and centrifuged for 10 min at 3, 2 gravity. The resulting supernatant was discarded and the bacteria was resuspended in 5 mL of phosphate-buffered saline (PBS) with a pH of 7.5 (Sigma-Aldrich, St. Louis) and mixed gently by vortexing for 10 sec.

We used DCT to test the antibacterial properties of the cements. The antimicrobial susceptibility profiles were determined by disk diffusion agar method according to CLSI M100-S12 protocols (2005). In each sterilized Petri dish (20100 mm), a base layer containing 15 mL of blood agar mixed with 100 μL of inoculum was prepared. After the solidification of culture medium, wells measuring 7 mm in diameter were made in each plate and the testing materials were transferred to wells. Two wells were served as the positive control without the tested cements. Plates were incubated at 37°C for 48 h and after that, diameters of zones of inhibition produced around the specimens were measured at three different points. The size of inhibition zones was calculated through subtracting the diameter of specimen (7 mm) from the average of three measurements of the halo. All measurements were performed twice by the same blinded operator. Antibacterial tests were repeated 5 times to confirm the homogeneity of the results. Moreover, diameters of zones of inhibition produced around specimens were measured after the reincubation of plates at 37°C for 5 days.

**Table 2:** The mean values and standard deviations of the inhibition zones and CFU for each material (mean ± SD).

Material	***S. mutans*** ** (inhibition zones)**		***C. albicans *** **(CFU)**	
	Day 2	Day 7	Day 2	Day 7
GIC (A)	22.80 ± 1.30	21.20 ± 1.30	412.0 ± 11.51	677.0 ± 37.35
GIC (B)	14.0 ± 0.70	13.40 ± 0.54	392.0 ± 11.78	691.40 ± 14.20
GIC (C)	12.40 ± 0.54	11.0 ± 0.70	476.80 ± 5.71	708.80 ± 14.46
ZPC (A)	12.20 ± 1.78	11.60 ± 0.89	389.80 ± 8.98	695.40 ± 14.02
ZPC (B)	11.40 ± 0.89	11.20 ± 0.44	386.80 ± 6.72	692.80 ± 11.88
ZPC (C)	9.40 ± 0.54	8.20 ± 0.44	401.60 ± 5.94	699.0 ± 10.39

*Antifungal activity test*

*Candida albicans *ATCC 10231 was placed on Sabouraud dextrose agar and incubated at 37^°^C for 24 h. After this period, a suspension was prepared in sterile saline solution (0.85% NaCl) with the aid of a spectrophotometer (CE 2501, CECIL Instruments, Series 2000, Cambridge, England), adopting the optical density of 450 nm.

The samples of cements were transferred to tubes containing 3 mL of Sabouraud Dextrose broth (Merck, Darmstadt, Germany). Then, 0.5 mL of the standardized *C. albicans *suspension was inoculated in each tube. Tubes were incubated at 37°C for 48 h. Tubes without any specimen of the tested materials were incubated as positive controls. After the incubation period, each initial suspension was diluted 10, 100 and 1,000 times in sterile saline solution, and 0.1 mL of each suspension was plated in duplicate on Sabouraud Dextrose agar (Merck, Darmstadt, Germany) and incubated in 37^°^C for 48 h. After this period, the number of colony-forming units per milliliter (CFU/mL) was obtained. Similar procedure was performed after 7 days of incubation.

*Statistical analysis*

The mean diameter of inhibition zone values for each material was used for statistical analysis by using General Linear Models to compare the inhibition zones of bacteria around each cement. Tukey’s studentized post-hoc tests were performed to identify the differences between the cements, the level of significance set at p < 0.001. A Tukey’s complementary test was also used to determine if there was a significant difference between the inhibitory effects of 2 and 7 days specimens (p < 0.001).

## Results and Discussion

The mean values and standard deviations of the inhibition zones and CFU for each material according to the bacteria strain, at different days, are shown in Table 2. There was a significant difference between the type of cements, brand of cements, time of evaluation and the antibacterial activity (Table 3). The antibacterial property of glass ionomer was more than zinc phosphate but antifungal activity was less than zinc phosphate in 2 days. However, there was no significant difference in antifungal activity in 7 days. A reduction in the measured inhibition zones was observed in all samples over 7 days that was not significant.

**Table 3:** Effect of brand of cement and time of evaluation on the antibacterial activity

Material	**Source**	***S. Mutans*** ** (inhibition zones)**					***C. Albicans *** **(CFU)**			
		**df**	**Mean Square**	**F**	**Sig.**	**df**		**Mean Square**	**F**	**Sig.**
GIC	Cement. Company	2	198.86	357.96	0.000	2		5962.822	21.954	0.000
	Timing	2	1253.60	2.256E3	0.000	2		365025.622	1.344E3	0.000
	Cement. timing	4	50.06	90.120	0.000	4		2548.022	9.382	0.000
ZPC	Cement. Company	2	18.022	29.491	0.000	2		237.222	2.016	0.148
	Timing	2	570.556	933.636	0.000	2		475126.022	4.038E3	0.000
	Cement. timing	4	4.822	7.891	0.000	4		61.456	0.522	0.720

Caries prevention in patients using orthodontic appliances is reliant on the control of dental plaque. However, many patients do not take care of their oral hygiene perfectly. Consequently, antibacterial properties of orthodontic band cements are desirable. Glass ionomer cement presents approving and essential properties such as biocompatibility to dental pulp, ability of chemical bonding to enamel and dentin and fluoride releasing, which can play an important role in the inhibition of bacteria growth and caries progression (21-24). Different *in-vitro* methods have been used to study the antibacterial activity of dental materials. Boeckh* et al.* throughout their experiments using strains of *S. mutans*, showed the important role of this microorganism in caries etiology (18).

The methodology applied in this research was based on DCT to verify the inhibition zone of materials evaluated and focused on the standardization of the experimental conditions, in particular in relation to the specimens’ dimensions and microorganism suspension. According to the results, all glass ionomer cements evaluated the inhibited bacterial growth, but with differences according to the material. The differences in growth inhibition between these cements may be related to their inherent potency and to different solubilities (16).

Yap and others reported that there was no antibacterial activity despite the presence of fluoride in the agar around the set materials (25). We found that all three GICs completely inhibited the growth of *S. mutans*. This effect lasted for at least one week. The most credible cause of the reduced bacterial growth after direct contact with the GIC is fluoride release from this material combined with a pH fall around the material as described elsewhere (26-27). The concentration of fluoride in a specific dental material does not reflect its rate of release. In consequence, the antibacterial properties of glass ionomer cements are different from one material to another. From a clinical point of view, the fluoride release of the GICs may drop significantly with long-term usage as reported in other studies (28-29). Our information recommends that further studies are required to examine the levels of fluoride release and the effects of GICs on complex biofilms. Furthermore, additional improvement of orthodontic cements is extremely important for displaying the long-lasting antibacterial properties together with the fluoride release.

In this study, the three commercial zinc phosphate cements showed an antibacterial activity. The antibacterial potential of these cements could be due to their low pH in the first min after the mixing and their ability to release ions that inhibit the growth of caries-related bacteria (30-32). The growth of *S. mutans *colonies is significantly decreased at low pH as described elsewhere (33). In this study, the inhibition of *S. mutans *growth was detectable with 2 days and 7 days aged cements and there was no significant difference between the antibacterial activities in these two days, so, indicating the unchanged effect of material pendant 1 week that was in agreement with other studies (34-35). The increase of pH after setting the material could explain the reduced antibacterial action of the phosphate cements during the time. Other studies have also demonstrated the inhibition of *S. mutans *growth due to ZPCs, followed by decreased antibacterial activity over the time due to the lower ion release levels (36). In the present study, we observed that the glass ionomer cements had significantly more antibacterial effect in comparison with the zinc phosphate cements. Other studies have also reported the most antibacterial properties of GICs between different cements (37-38). This could be explained by the combined effect of low pH of GICs and their fluoride-leaching capabilities (33). In all experiments, the antibacterial activity measured with cements A and B was greater than that of cement C, which is estimated to be correlated with the higher zinc or fluoride release rates observed with the formers.

Few studies of Oral *Candida *spp. level control have been reported in the related literature. In our study, counts of *C. albicans *after 48 h were lower and statistically different (p < 0.001) in the GIC group in relation to the control groups. But no differences were observed between GIC and control groups at 7 days. Another study reported that GICs had no antifungal effect in 2 days (39). We detected that zinc phosphate cements had significantly more antifungal effects, after 48 h, compared with the glass ionomer cements. The findings of the present study showed that the cements A and B were more effective in reducing *Candida *spp. colony counts than the cement C.

Based on the results of the present study, it can be concluded that all the evaluated cements displayed some antimicrobial activity. This antimicrobial activity was cement-and time-dependent. GIC (A) and GIC (B) were the most active antibacterial cements and ZPC (A) and ZPC (B) were the most active antifungal cements. Combined with the mechanical and biological properties, these differences should be taken into account when one is choosing cement for orthodontic clinical use.
